# An Improved FastICA Method for Fetal ECG Extraction

**DOI:** 10.1155/2018/7061456

**Published:** 2018-05-17

**Authors:** Li Yuan, Zhuhuang Zhou, Yanchao Yuan, Shuicai Wu

**Affiliations:** College of Life Science and Bioengineering, Beijing University of Technology, Beijing, China

## Abstract

**Objective:**

The fast fixed-point algorithm for independent component analysis (FastICA) has been widely used in fetal electrocardiogram (ECG) extraction. However, the FastICA algorithm is sensitive to the initial weight vector, which affects the convergence of the algorithm. In order to solve this problem, an improved FastICA method was proposed to extract fetal ECG.

**Methods:**

First, the maternal abdominal mixed signal was centralized and whitened, and the overrelaxation factor was incorporated into Newton's iterative algorithm to process the initial weight vector randomly generated. The improved FastICA algorithm was used to separate the source components, selected the best maternal ECG from the separated source components, and detected the R-wave location of the maternal ECG. Finally, the maternal ECG component in each channel was removed by the singular value decomposition (SVD) method to obtain a clean fetal ECG signal.

**Results:**

An annotated clinical fetal ECG database was used to evaluate the improved algorithm and the conventional FastICA algorithm. The average number of iterations of the algorithm was reduced from 35 before the improvement to 13. Correspondingly, the average running time was reduced from 1.25 s to 1.04 s when using the improved algorithm. The signal-to-noise ratio (SNR) based on eigenvalues of the improved algorithm was 1.55, as compared to 0.99 of the conventional FastICA algorithm. The SNR based on cross-correlation coefficients of the conventional algorithm was also improved from 0.59 to 2.02. The sensitivity, positive predictive accuracy, and harmonic mean (*F*1) of the improved method were 99.37%, 99.00%, and 99.19%, respectively, while these metrics of the conventional FastICA method were 99.03%, 98.53%, and 98.78%, respectively.

**Conclusions:**

The proposed improved FastICA algorithm based on the overrelaxation factor, while maintaining the rate of convergence, relaxes the requirement of initial weight vector, avoids the unbalanced convergence, reduces the number of iterations, and improves the convergence performance.

## 1. Introduction

Electrocardiogram (ECG) is an important tool used by physicians for identifying abnormalities in the human heart activity [1]. Similarly, fetal ECG signal can also reflect electrophysiological activity of the fetal heart. Physicians can detect in time fetal abnormalities during fetal development through the fetal ECG waveform analysis, such as fetal distress and intrauterine hypoxia. In addition, a small number of abnormal fetal ECG waveforms are also a manifestation of congenital heart disease, for which early measures can be taken to reduce neonatal morbidity and mortality.

Currently, there are two ways for obtaining fetal ECG. One is the invasive scalp electrode method, which can directly measure the pure fetal ECG signal. However, it can only detect fetal ECG signal during the time of birth, and it is invasive so it may cause harm to the mother and the fetus. The other method is noninvasive, abdominal electrode method. The signals from the abdominal body surface are collected by placing an electrode patch in the abdomen of the mother, which allows for long-term monitoring during pregnancy without harming the mother or the fetus. However, the signals from maternal abdomen surface are very complex, which not only contain weak fetal ECG and maternal ECG but also include the mother's respiratory noise, frequency interference, and other signals [2]. In particular, the magnitude of the maternal ECG detected in the abdomen is about 2–10 times that of the fetal ECG [3], which makes the extraction of fetal ECG difficult. Therefore, it is necessary to develop a noninvasive method that can extract fetal ECG effectively.

At present, fetal ECG extraction algorithms mainly include adaptive filtering [4, 5], wavelet analysis [6], matched filtering [7], blind source separation [8], independent component analysis (ICA) [9], neural network [10, 11], and singular value decomposition (SVD) [12]. Among these methods, ICA can separate the source signals from the mixed signals under the assumption that the source signals are statistically independent of each other, without needing any information regarding the source signals or the mixed matrix. Therefore, ICA is considered as a promising method for extracting fetal ECG. In recent years, researchers have proposed many improved ICA algorithms, which can separate non-Gaussian signals. Among them, because of its fast convergence, the fast fixed-point algorithm for independent component analysis (FastICA) [13] has been widely used in the extraction of fetal ECG. However, the FastICA algorithm is sensitive to the initial weight vector, and different initial weight vectors may lead to different convergence performances of the algorithm.

In this paper, an improved FastICA method was proposed to solve the above problem. By incorporating an overrelaxation factor into the iterative algorithm, the initial weight vector generated randomly can be relaxed. By choosing the appropriate overrelaxation factor, the iterative algorithm with slower convergence rate can converge, and the divergent iterative algorithm may become convergent.

## 2. Methods

### 2.1. FastICA Algorithm

FastICA is a fixed-point iterative algorithm, minimizing mutual information between estimated components [14]. Separation of independent components is accomplished when the maximum of non-Gaussianity is attained. There are different kinds of FastICA, including those based on kurtosis, based on the maximum likelihood, and based on the maximum negentropy (MNE), and so forth. In this paper, the MNE-based FastICA algorithm was used, which took the maximization of negentropy as a search direction and extracted each independent source signal in turn.

Before using the FastICA algorithm, the observed signal *X* was centralized and whitened. The mean removal process was conducted to subtract the mean vector of the signal from the observed signals so that the observed signal became zero mean, simplifying the FastICA algorithm. The observation signal was whitened using the principal component analysis (PCA) whitening algorithm so that the components after the whitening were uncorrelated. The purpose of the FastICA algorithm based on fixed-point iterative structure was to make *y* = *w*^*T*^*x* have the maximum non-Gaussianity, where *w* was a row of the separation matrix *W*. The objective function was set as

(1) $$\begin{array}{c} J\left(\;y\;\right)\approx {\left\{\;E\left[\;G\left(\;y\;\right)\;\right]-E\left[\;G\left(\;v\;\right)\;\right]\;\right\}}^{2}, \end{array}$$

where *E*[·] was the expectation operator and *v* was a Gaussian random variable with zero mean and unit variance. It was assumed that *y* also had zero mean and unit variance. *G*(·) was a nonquadratic function.

According to the Kuhn-Tucker condition, the optimization of *E*{*G*(*w*^*T*^*x*)} could be obtained by (2) under the constraint of *E*{(*w*^*T*^*x*)^2^} = ‖*w*‖^2^ = 1:

(2) $$\begin{array}{c} E\left\{\;xg\left(\;w^{T}x\;\right)\;\right\}-\beta w=0, \end{array}$$

where *β* was a constant and could be obtained by *β* = *E*{*w*_0_^*T*^*xg*(*w*_0_^*T*^*x*)}, where *w*_0_ was the initial value of *w* and *g*(·) was a nonlinear function, which was the derivative of *G*(·). We chose the nonlinear function *g*(*y*) = *y*^3^. The Newton iterative method was employed to solve (2). The left part of (2) was denoted as *F*(*w*), and the Jacobian matrix *JF*(*w*) was

(3) $$\begin{array}{c} JF\left(\;w\;\right)=E\left\{\;xx^{T}g^{\prime }\left(\;w^{T}x\;\right)\;\right\}-\beta I \end{array}$$

In order to simplify the computation of the inverse of the matrix, (3) was approximated. Because the data were whitened, (3) could be simplified as

(4) ExxTg′wTx≈ExxTEg′wTx=Eg′wTxI

The Jacobin matrix was a diagonal matrix, and its inverse matrix could be simply calculated. Similarly, replace the value of *w*_0_ with the current value of *w* for the constant *β*. Therefore, we could obtain the approximated Newton iterative formula as follows:

(5) $$\begin{array}{c} w_{k+1}=w_{k}-\frac{\left[E\left\{xg\left(w_{k}^{T}x\right)\right\}-\beta w_{k}\right]}{\left[E\left\{g^{\prime }\left(w_{k}^{T}x\right)\right\}-\beta \right]}, \end{array}$$

where *β* = *E*{*w*^*T*^*xg*(*w*^*T*^*x*)} and *w*_*k*+1_ represented the updated value of *w*_*k*_. In order to improve the stability of the algorithm, *w* was normalized by *w*_*k*+1_ = *w*_*k*+1_/‖*w*_*k*+1_‖ after an iteration. To simplify (5), we obtained an iterative formula of simplified FastICA algorithm:

(6) $$\begin{array}{c} w_{k+1}=E\left\{\;xg\left(\;w_{k}^{T}x\;\right)\;\right\}-E\left\{\;g^{\prime }\left(\;w_{k}^{T}x\;\right)\;\right\}w_{k}. \end{array}$$

### 2.2. FastICA Algorithm Improvement

#### 2.2.1. Selection of Overrelaxation Factor

In order to address the problem that FastICA is sensitive to the initial weight vector, we introduced an overrelaxation factor *α*_*k*_ into the iterative algorithm. If *F*(*w*_*k*_) is guaranteed to have a decreasing property for a given norm, (7) was satisfied:

(7) Exgwk+1Tx−βwk+1<ExgwkTx−βwkk=0,1,…,

that is,

(8) $$\begin{array}{c} \underset{\alpha_{k}}{\min}\left\|\;F\left(\;w_{k}-\frac{\alpha_{k}F\left(w_{k}\right)}{JF\left(w_{k}\right)}\;\right)\;\right\| \end{array}$$

By introducing the overrelaxation factor *α*_*k*_, it was guaranteed that *F*(*w*_*k*_) can enter the convergence area of the Newton iteration algorithm from a certain value of *w*_*k*_, so as to ensure that the algorithm can achieve the convergence effect under any circumstance.

There are many methods for choosing the overrelaxation factors, such as the golden section method and step-by-step experimental method. In this paper, we used step-by-step experiment to obtain the optimal overrelaxation factor. The value of *α*_*k*_ is 1 + 1/*N*, 1 + 2/*N*,…, 1 + (*N* − 1)/*N*, where *N* = 100. All the values of *k* that satisfied (9) were recorded. Among these values of *k*, the one that satisfied (8) was found and denoted as *p*. Then, *α*_*p*_ would be the optimal overrelaxation factor *α*.

(9) $$\begin{array}{c} {\left\|\;F\left(\;w_{k}-\alpha_{k}\Delta w_{k}\;\right)\;\right\|}^{2}<{\left\|\;F\left(\;w_{k}\;\right)\;\right\|}^{2}, \end{array}$$

where Δ*w*_*k*_ = *F*(*w*_*k*_)/*JF*(*w*_*k*_).

The selection of the overrelaxation factor *α* included the following steps.

**Step 1 .**

Set the initial value *N* = 100, *k* = 1, and create an empty vector *v*.

**Step 2 .**

*α*_*k*_ = 1 + *k*/*N*.

**Step 3 .**

If the value of *k* satisfies (9), then *k* is used to calculate the target function TF, TF = ‖*F*(*w*_*k*_ − *α*_*k*_*F*(*w*_*k*_)/*JF*(*w*_*k*_))‖, and the result of TF is added to *v*.

**Step 4 .**

*k* = *k* + 1.

**Step 5 .**

If *k* < 100, repeat Steps 2–4. Otherwise, the value of *k* corresponding to the smallest value of TF in the vector *v* is found and denoted as *p*. Then, *α*_*p*_ is the optimal overrelaxation factor *α*.

#### 2.2.2. Improved FastICA

The convergence performance of the FastICA algorithm was affected by the initial weight vector. Since the initial weight vector of the FastICA algorithm was selected randomly, the efficiency of each iteration was different due to different initial weight vector. The obtained independent components would also be slightly different. Although it was also possible to select the principal component obtained during the whitening process as the initial weight vector, the algorithm easily converged to the initial value of the whitening. The FastICA algorithm should relax the initial weight vector requirements, that is, to achieve convergence in a wide range.

In order to improve the FastICA algorithm with respect to the initial weight vector *w*_0_, Xu et al. [15] proposed the incorporation of relaxation factor *α*_*k*_ (low relaxation factor 0 < *α*_*k*_ < 1) into the FastICA iteration. Because the negative gradient direction was the fastest declining direction, the gradient value was chosen as the optimal relaxation factor *α*_*k*_ in [15]. However, the computational of the relaxation factor was of high complexity [15]. In this paper, the overrelaxation factor *α*_*k*_ (1 < *α*_*k*_ < 2) was introduced into the FastICA iteration to deal with the initial weight vector generated randomly, and the requirement of the initial weight vector of the FastICA algorithm was relaxed. Compared with the low relaxation iteration [15], the proposed overrelaxation iteration converged much faster and was of low computational complexity.

In this paper, we introduced the overrelaxation factor into the iteration of FastICA algorithm. The value of *α* was calculated according to Section 2.2.1. The iteration of *w*_*k*+1_ became as follows:

(10) w′k+1=ExgwkTx+αEg′wkTxwkw′′k+1=ExgwkTx+Eg′w′k+1Txwkwk+1=w′′k+1w′′k+1

### 2.3. Fetal ECG Extraction by Improved FastICA

To achieve long-term monitoring of pregnant women during the perinatal period, noninvasive abdominal methods were preferred to collect mixed signals from the maternal abdomen. The collected mixed signals contained not only maternal ECG and fetal ECG, but also some noises. Before using the improved FastICA algorithm to extract fetal ECG, the observed signals were preprocessed. The third-order low-pass Butterworth filter was used to estimate the baseline signal of each channel, and the low-pass filter cut-off frequency was 5 Hz. The baseline drift removal signal was obtained by subtracting the baseline signal from the observed signal. Then, the processed signal was centralized and whitened to make the signal uncorrelated, thus reducing the complexity of the algorithm. The improved FastICA was used to separate each component of the source signal. As the amplitude of the maternal ECG signal is 2–10 times that of the fetal ECG signal, the extracted fetal ECG signal usually contained some maternal ECG interference. In order to remove the maternal ECG interference, the maternal ECG signal channel was selected from the separated signal components, and R-wave detection was conducted on the maternal ECG signal. Then, the SVD method was used to remove the maternal ECG components in each channel to obtain clean fetal ECG without maternal ECG interference [16].

Fetal ECG extraction steps were as follows.

**Step 1 .**

Remove baseline drift and centralize the observed signal *X*, $\overset{-}{X}=X-E\{X\}$.

**Step 2 .**

$X=ED^{-1/2}E^{T}\bar{X}$.

**Step 3 .**

Initialize random vector *w*_0_, and set the error of convergence 0 < *ε* < 1.

**Step 4 .**

According to Section 2.2.1, calculate overrelaxation factor *α*.

**Step 5 .**

According to (10), adjust *w*_*k*+1_.

**Step 6 .**

Calculate *w*_*k*+1_, and normalize it.

**Step 7 .**

If |*w*_*k*+1_ − *w*_*k*_| > *ε*, the algorithm does not converge; return to Step 5 until the algorithm converges.

**Step 8 .**

Using the resulting separation matrix *w*, all the estimates of the source signal *y*_*i*_ are obtained. The maternal ECG component is selected, and R-wave detection is performed.

**Step 9 .**

Use SVD to remove the maternal ECG components in each channel to get a clean fetal ECG signal.

## 3. Fetal ECG Extraction Algorithm Evaluation

The signal-to-noise ratio (SNR) and statistical evaluation were used to compare the performances of the proposed algorithm and the conventional FastICA algorithm in terms of fetal ECG extraction performance.

### 3.1. Signal-to-Noise Ratio Evaluation

The signal-to-noise ratio (SNR) based on eigenvalues and the SNR based on cross-correlation coefficients proposed by Outram [17] were used to evaluate the performance of the proposed algorithm and the conventional FastICA algorithm.

First, R-wave detection of the extracted fetal ECG was performed. Each R-wave was taken as a reference to cut off *M* signal segments whose length is *N*. Each signal segment contained a complete QRS wave. Then, the *M* signal segments were used to form a matrix *U*_*N∗M*_, and the normalization of each column of the matrix with zero mean and unit variance was performed. The SNR based on eigenvalues was defined as follows:

(11) $$\begin{array}{c} {SNR}_{Eig}=\sqrt{\frac{\lambda_{max}}{sum\left(\lambda \right)-\lambda_{max}}}, \end{array}$$

where *λ* was the *M* eigenvalues of the matrix *U*^*T*^*U* and *λ*_max_ was the maximum of the eigenvalues of the matrix *U*^*T*^*U*.

(2) The SNR based on cross-correlation coefficients is defined in

(12) $$\begin{array}{c} SNR_{RMS}=\sqrt{\frac{\eta }{1-\eta }}, \end{array}$$

where *η* = (2/*M*(*M* − 1))∑_*i*=0_^*M*−2^∑_*k*=*i*+1_^*M*−1^*f*(*i*)^*T*^*f*(*k*), where *f* is the fetal ECG signal that contains intact QRS waves.

### 3.2. Statistical Evaluation

The performance of the methods was evaluated on the total length of fetal QRS signals, using sensitivity (Sens), positive predictive accuracy (PPA) [18], and harmonic mean (*F*1) [19]:

(13) Sens=TPTP+FNPPA=TPTP+FPF1=2PPA·SensPPA+Sens=2·TP2·TP+FN+FP,

where TP, FP, and FN are the number of true positive (correct detection of fetal ECG QRS complexes), false positive (falsely detected absence of R-peak), and false-negative (false fetal ECG QRS complex detection), respectively. *F*1 is the overall probability that the fetal ECG QRS complex is correctly detected and can be used as a measure of the accuracy of the proposed method. The Pan and Tompkins algorithm [20] was used to detect the fetal QRS in this work.

## 4. Results

The improved FastICA algorithm was applied to fetal ECG signal extraction. The algorithm was validated using the clinical database Abdominal and Direct Fetal Electrocardiogram Database (ADFECGDB) [21]. The data were collected from five pregnant women of childbirth at 38–41 weeks of gestation. Each record contains four signals obtained from the maternal abdomen and one acquired directly from the fetal head at a sampling rate of 1 kHz and a collection time of 5 minutes.

**Figure 1:**
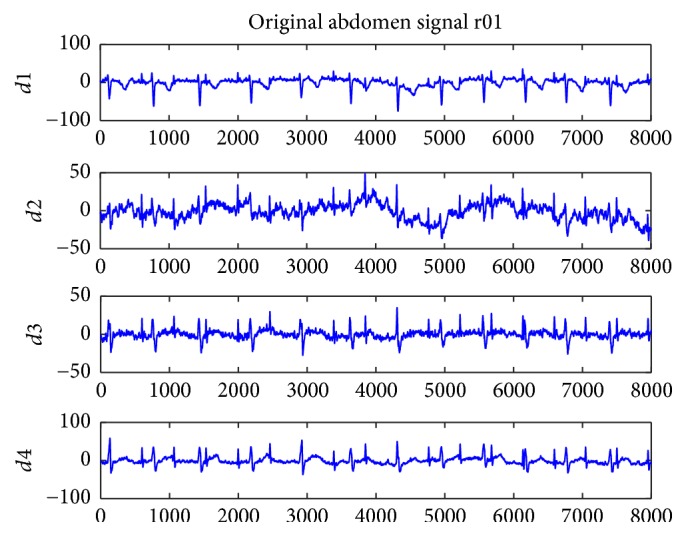
The original abdominal signal r01.

**Figure 2:**
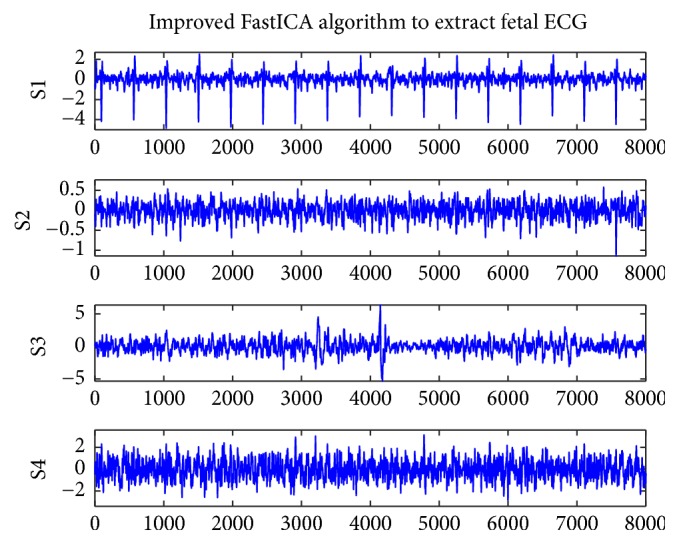
Improved FastICA algorithm to extract fetal ECG for r01.

**Figure 3:**
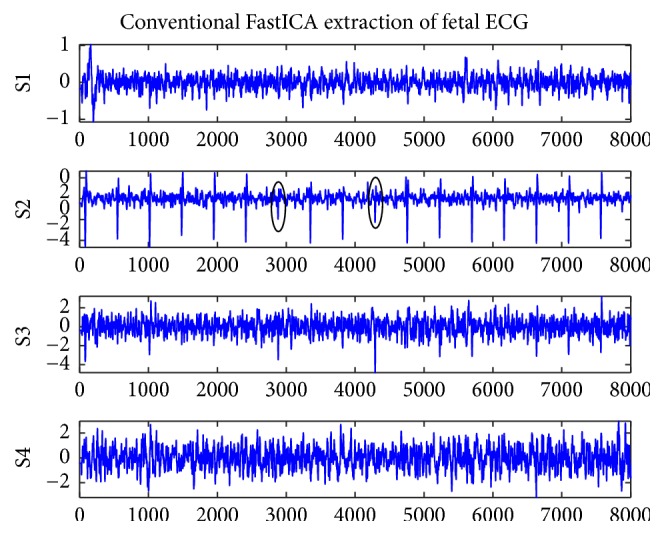
Conventional FastICA extraction of fetal ECG for r01.

Figure 1 shows the original record of the maternal abdomen signal database named r01. Figures 2 and 3 represent the residual signals after the cancelling of the maternal ECG component.  Figure 2 shows the four ECG components of r01 extracted by the improved FastICA algorithm, where S1 is the extracted fetal ECG signal.  Figure 3 shows the four ECG components of r01 extracted by the conventional FastICA algorithm, where S2 is the extracted fetal ECG signal. The amplitudes of R-waves (denoted by black circles in S2 of Figure 3) are small, which may render the R-waves undetected.

**Figure 4:**
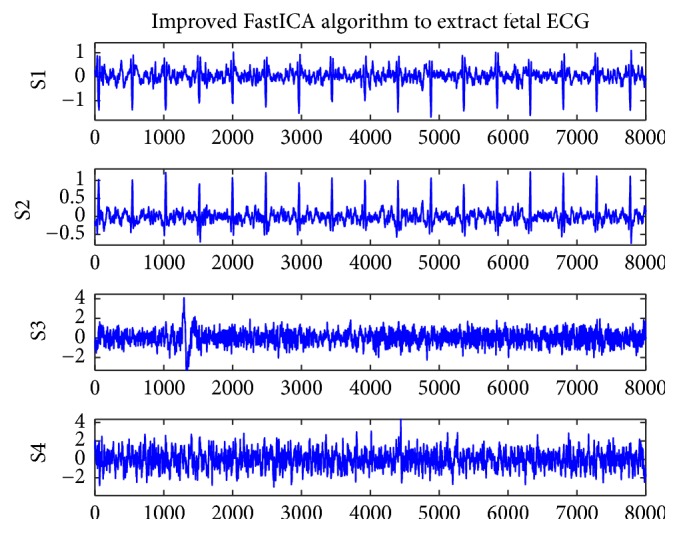
Improved FastICA algorithm to extract fetal ECG for r04.

**Figure 5:**
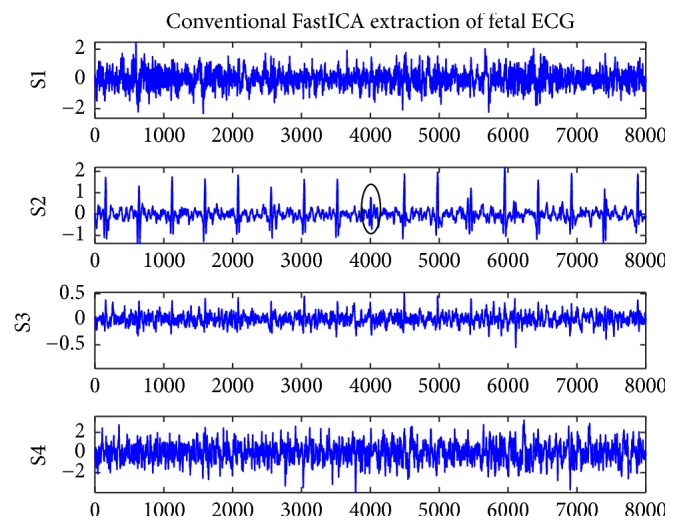
Conventional FastICA extraction of fetal ECG for r04.

Figure 4 shows the four ECG components of r04 extracted by the improved FastICA algorithm, where both S1 and S2 contain fetal ECG signals. It can be seen that the proposed algorithm can extract clean fetal ECG from maternal abdominal mixed signals.  Figure 5 shows the four ECG components of r04 extracted by the conventional FastICA algorithm, where S2 is the extracted fetal ECG signal. The amplitude of the 9th R-wave (denoted by black circles in S2 of Figure 5) is small, which may render the R-wave undetected.

**Figure 6:**
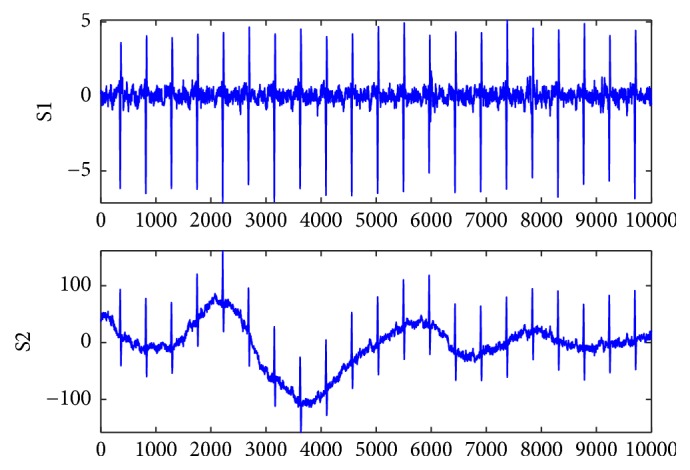
Improved FastICA algorithm for extraction of fetal ECG (S1) and golden standard (S2) for r01.

**Figure 7:**
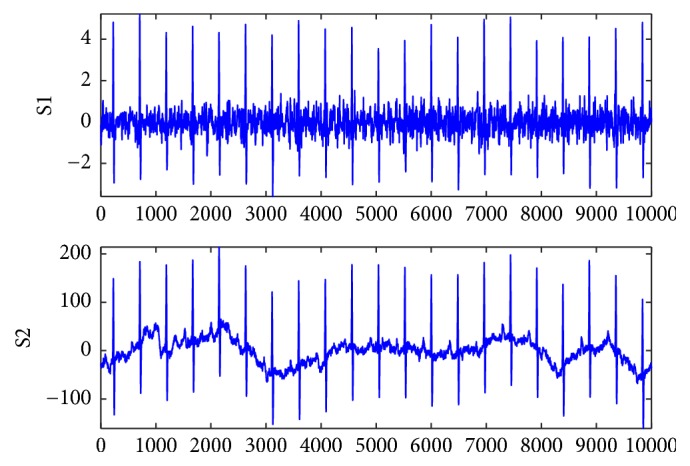
Improved FastICA algorithm for extraction of fetal ECG (S1) and golden standard (S2) for r08.

In Figures 6 and 7, the S1 signals are the fetal ECG signal extracted from the abdomen signals of r01 and r08 using the proposed algorithm, respectively; the S2 signals are the fetal ECG signal provided the database as golden standard. It can be found that the proposed algorithm can extract a clean fetal ECG without loss of R-waves.

Table 1 shows the number of iterations and computational time of the conventional and improved FastICA algorithms for separating four source signal components. As the number of iterations of the two algorithms is related to the initial weight vector *w*_0_, each algorithm ran 10 times. The average number of iterations of the conventional and proposed FastICA algorithms was 35 and 13, respectively, and the computational time was 1.25 s and 1.04 s, respectively. The number of iterations and computational time were decreased and the convergence rate was improved when using the improved FastICA algorithm.

Table 2 shows the SNR of the conventional and improved FastICA algorithms. Both the SNR based on eigenvalues and the SNR based on cross-correlation coefficients of the proposed algorithm were better than that of the conventional FastICA algorithm.

There was a one-channel annotated fetal ECG in ADFECGDB that can accurately provide the location of the fetal heart. As shown in Table 3, the proposed method correctly detected 3171 (TP) actual QRS complexes, falsely detected 32 (FP) extra QRS complexes, and missed 20 (FN) actual QRS complexes. The *F*1 of our proposed method was 99.19% while the *F*1 of the conventional FastICA method was 98.78%. It is demonstrated that the method proposed in this paper performs more favorably than the conventional FastICA method in statistical evaluation.

## 5. Discussion

The FastICA has been widely used in the extraction of fetal ECG because of its fast convergence rate. However, the FastICA algorithm is sensitive to the initial weight vector, and different initial weight vectors may lead to different convergence performances of the algorithm. So, in this paper, we introduced an overrelaxation factor, which can reduce the dependence of the FastICA algorithm on the initial weight vector and improve the convergence speed. The clinical database Abdominal and Direct Fetal Electrocardiogram Database (ADFECGDB) was selected to test the improved algorithm and the conventional FastICA algorithm. Figures 2 and 3 represent the residual signals obtained by maternal ECG component cancellation from the source components separated by the improved and conventional FastICA algorithms, respectively. It can be found that the proposed algorithm can extract a clean fetal ECG without loss of R-waves. We selected the four-channel AECG signal and compared the number of iterations and the computational time of the two algorithms. It can be seen from Table 1 that the average number of iterations of the improved algorithm is 13 and the average computational time is 1.04 s, significantly better than the conventional FastICA algorithm. As can be seen from Table 3, the improved algorithm only yielded 32 FP fetal heartbeat detections and 20 FN fetal heartbeat detections based on ADFECGDB, which contained a total of 3191 fetal heartbeats. The *F*1 of our improved method was 99.19% while the *F*1 of the conventional FastICA method was 98.78%. These results imply that the method improved in this paper is superior to the conventional FastICA method in statistical evaluation.

This study has a limitation. The improved algorithm and the conventional FastICA algorithm were only tested on the clinical database ADFECGDB. In future work, more clinical data may be used to further evaluate the performance of the improved FastICA algorithm.

## 6. Conclusions

In this paper, the FastICA algorithm based on the improved overrelaxation factor was proposed to extract fetal ECG. By incorporating an overrelaxation factor into the iterative algorithm, the initial weight vector generated randomly can be relaxed. ADFECGDB was used to assess the performance of the proposed algorithm and the conventional FastICA algorithm. Compared with the conventional FastICA algorithm, the proposed method not only had a fewer number of iterations but also performed better in SNR, Sens, PPA, and *F*1 metrics.

## Figures and Tables

**Table 1:** Number of iterations and running time of the improved and conventional FastICA algorithms.

No.	Improved FastICA		Conventional FastICA	
	Number of iterations	Running time (s)	Number of iterations	Running time (s)
1	16	1.03	34	1.73
2	14	1.06	36	1.20
3	12	1.03	28	1.21
4	12	1.03	32	1.18
5	14	1.02	36	1.23
6	12	1.02	34	1.18
7	14	1.06	34	1.19
8	13	1.05	38	1.20
9	12	1.06	42	1.17
10	12	1.01	36	1.18
Average	13	1.04	35	1.25

**Table 2:** Signal-to-noise ratio of the improved and conventional FastICA algorithms.

	Improved FastICA algorithm	Conventional FastICA algorithm
SNR_Eig_	1.55	0.99
SNR_RMS_	2.02	0.59

**Table 3:** Statistical evaluation of the improved and conventional FastICA algorithms.

Method	TP	FP	FN	Sens (%)	PPA (%)	*F*1 (%)
Improved FastICA	3171	32	20	99.37	99.00	99.19
Conventional FastICA	3160	47	31	99.03	98.53	98.78

## Data Availability

The data used to support the findings of this study are available at https://physionet.org/physiobank/database/adfecgdb/.
